# Supplement

**Published:** 1867-07

**Authors:** 


### Editorial.

***Supplement.***

The large mass of material crowding upon THE JOURNAL, necessitates publication of the doings of the State Medical Society by means of a supplementary form of sixteen pages. Even with this addition, we find it impossible to publish the transactions of several Societies, which have been kindly forwarded. We are particularly melancholy in the reflection that our own article, detailing the doings of the American Medical Association, has to wait a little longer in the editorial pigeon holes. Our philanthropic and humanitarian sensibilities are touched to think that the professional leviathans, who only come to the surface to spout at this great occasion, cannot be aired by THE JOURNAL just yet. Nevertheless, our bowels of compassion have been so stirred within us by perusal of the doings that we almost think it providential that our readers are not endangered likewise in this threatening cholera season. But patience! What "A. B. P." did, and what the "Apostle" and "Father" said in explanation, shall they not be immortalized, like names "writ in water," in the TRANSACTIONS?

